# Validation of Multi-Residue Method for Quantification of Antibiotics and NSAIDs in Avian Scavengers by Using Small Amounts of Plasma in HPLC-MS-TOF

**DOI:** 10.3390/ijerph17114058

**Published:** 2020-06-06

**Authors:** Pilar Gómez-Ramírez, Guillermo Blanco, Antonio Juan García-Fernández

**Affiliations:** 1 Toxicology and Forensic Veterinary Service, Dept. of Socio-Health Sciences, Faculty of Veterinary, University of Murcia, Campus de Espinardo, 30100 Murcia, Spain; ajgf@um.es; 2 Toxicology and Risk Assessment Group, Biomedical Research Institute of Murcia (IMIB-Arrixaca), University of Murcia, Campus de Espinardo, 30100 Murcia, Spain; 3 Department of Evolutionary Ecology, Museo Nacional de Ciencias Naturales, CSIC, José Gutiérrez Abascal 2, 28006 Madrid, Spain; gblanco@mncn.csic.es

**Keywords:** veterinary pharmaceuticals, risk assessment, analysis, vultures, drug mixtures

## Abstract

Pharmaceuticals are still considered emerging pollutants affecting both aquatic and terrestrial ecosystems. Scavenging bird species may be exposed to veterinary drugs when they feed on livestock carcasses provided at supplementary feeding stations, as these are often stocked with ailing and/or recently medicated animals. Because those animals may be a source of several different pharmaceutical compounds, analytical methods to evaluate residue levels and exposure potential should enable detection and quantification of as many different compounds as possible, preferably from small sample volumes. Four different extraction methods were tested to conduct HPLC-MS-TOF analysis of some of the most common veterinary drugs used in livestock in Spain. The method deemed most viable was a simple extraction, using methanol and 100 µL of plasma, that allowed quantification of seven antibiotics (tetracycline, oxytetracycline, ciprofloxacin, enrofloxacin, nalidixic acid, trimethoprim, sulfadiazine) and five nonsteroidal anti-inflammatory drugs (NSAIDs) (meloxicam, flunixin, carprofen, tolfenamic acid, phenylbutazone). The method was then applied to analysis of 29 Eurasian griffon vulture (*Gyps fulvus*) nestling samples, wherein enrofloxacin and tolfenamic acid were most commonly detected (69% and 20%, respectively). To our knowledge, this is the first study including NSAIDs in the exposure assessment of different classes of veterinary pharmaceuticals in live avian scavengers.

## 1. Introduction

The use of pharmaceuticals leads to the constant release of bioactive substances into the environment. Although most of them are rapidly metabolized and degraded, there is a continuous input to the environment through different pathways, while other active substances are more persistent and even bioaccumulative [1]. As such, pharmaceuticals remain emerging pollutants of increasing concern worldwide [2]. Traditionally, the aquatic environment was considered the primary input source and conduit of these type of contaminants, mainly due to wastewater generated in urban, agricultural and livestock areas [3,4], but exposure within the terrestrial environment has recently attained increasing attention. Among the primary sources, intensive farming and the abandonment of carcasses from previously treated animals are considered some of the most important [5,6,7]. Due to their top position in trophic chains, the risk for exposure and accumulation of contaminants is generally high in predatory and scavenger species [8,9]. In the case of avian scavengers, both obligate and facultative, the risk for exposure to other (emerging) pollutants such as veterinary drugs can be increased when their diet is based on livestock carcasses provided in supplementary feeding stations (SFSs) [10,11,12,13].

Withdrawal periods, time that must elapse after the administration of a veterinary drug to livestock until its residues in animal products for human consumption are below the maximum residue limits (MRLs), are established according to the legally binding food safety standards to avoid risks to human health [11]. However, these food safety standards (withdrawal periods and MRLs) do not apply to animal health, and carcasses disposed in SFSs usually are from ailing and, in many cases, treated animals, so the concentrations of pharmaceuticals in carrion can still be quite high. Because these carcasses can be consumed by avian scavengers almost immediately after disposal [12], there is a high probability of exposure to veterinary pharmaceuticals and pathogens as a non-target consequence of supplementary feeding [6,13]. In fact, the ingestion of carrion from cows that had been previously treated with diclofenac, a nonsteroidal anti-inflammatory drug (NSAID), led to collapse of *Gyps* vulture population across South Asia during the 1990s [14], known as the “the Asian vulture crisis”, a paradigmatic case in wildlife toxicology. As a consequence, there is an increasing interest in the risk assessment of pharmaceuticals in scavenger species. To date, several compounds have been found in these species, including residues of antibiotics [13,15,16,17], antiparasitics [18] and other drugs identified in plasma and tissues as cause of death (e.g., pentobarbital [19,20], ketoprofen [21] and flunixin [19,22]).

For an adequate risk assessment of pharmaceuticals in avian scavengers, it should be taken into account that, considering their feeding habit, there is a real possibility that those species feeding on carcasses at SFSs are exposed to mixtures of pharmaceuticals. Among them, antibiotics and NSAIDs are some of the most commonly used in livestock [23]. Therefore, in this context, analytical methods for biological samples should ideally enable detection and quantification of as many relevant pharmaceutical compounds as possible. However, the chemical structure of veterinary pharmaceuticals is generally characterized as complex, with highly variable hydrosolubilities, low volatilization capacity, several ionizable functional groups (amphoteric molecules) and different pKa values [24]. Moreover, depending on the pH, these compounds may become neutral, cationic, anionic or zwitterionic. All these factors can influence analytical techniques for extraction and quantification [3]. This may explain the fact that published multiresidue analytical methods to quantify veterinary pharmaceutical levels in animal samples usually focus on a group of compounds (for a review, see [25]). Nevertheless, there is an increasing number of published methods for qualitative and quantitative analyses of veterinary drugs of different chemical classes in these complex matrices [26,27,28]. In addition, another challenge in veterinary drug residue analysis is that these contaminants usually occur in trace concentrations, frequently below limits of detection of most analytical instruments [25]. An added limitation occurs in free-living wild birds, where the available sample amount can be low for some species, which forces the miniaturization of the extraction methods and can lead to lower efficiencies. In general, the volume of collected blood should be less than 2% of the body weight of the animal in any 14-day period or no more than 1% at any one time [29,30]. In addition, biomonitoring studies often include the assessment of exposure to other environmental contaminants such as metals, agrochemicals or flame retardants in the same individuals [31]. In that case, at least 2.5 mL of blood will not be available for pharmaceutical analyses, as it is generally the minimum volume used for analysis of other contaminants ([32], see Electronic Supplementary Materials).

The aim of this study was to validate a multiresidual analytical technique to detect, in small plasma volumes, veterinary pharmaceuticals (seven antibiotics and five NSAIDs) commonly used in the treatment of livestock. To check the validity of the analytical method, plasma samples from Eurasian griffon vulture (*Gyps fulvus*) nestlings were analyzed. The Spanish vulture population represents a stronghold for this species in Europe, and it can be used as sentinel of pharma-pollution for other endangered species. This evaluation is paramount to avoid population crashes due to mortality directly caused by particular compounds, such as NSAIDs [14], or due to indirect impacts on health caused by antibiotics [13,15].

## 2. Materials and Methods

### 2.1. Biological Samples

Blood from healthy hens (*Gallus gallus domesticus*) from the Laboratory Animals section from University of Murcia, with authorization code CEEA 177/2015 was used for validation of the technique. These hens were only fed with nonmedicated feed, and they never were treated with pharmaceuticals. Blood was collected by puncturing the brachial vein with a 23 G needle and 5 mL syringe, using heparin as anticoagulant. To obtain plasma, blood samples were centrifuged at 3000 × *g* for 10 min. To check the validity of the technique, we used plasma of nestling Eurasian griffon vultures (*n* = 29) sampled from a breeding population in the Central Mountains range and associated canyons in Castilla y León, Central Spain, where a large and increasing vulture population nests on cliffs [33,34] and feeds in SFSs provided with pork and chicken carcasses [35,36]. Nests were accessed by climbing at the time that nestlings were feathered but not yet ready to fly (about 50–80 days old). The nestlings were ringed and measured, and blood samples (3–5 mL) were taken from the brachial vein (details in [37]). Ethical guidelines from the Spanish Royal Decree 1205/2005 on the protection of animals for experimentation and scientific research were followed, with permits from the Spanish Bird Ringing Centre (Permit Number: 530115) and the regional government of Castilla y Léon (Expedient Numbers: EP/CyL/282/2013–298/2016). Manipulation of vulture samples was performed following the best practice sampling and contaminant monitoring protocol for raptors developed by the ERBFacility COST-Action ([32], see link to Video 1). Blood samples were kept in tubes with heparin and transported under refrigeration conditions in polystyrene isothermal boxes to the laboratory. Every sample was centrifuged (3000× *g* for 10 min) in order to obtain plasma ([32], see link to Video 2) that was kept at −80 °C until the moment of its analysis.

### 2.2. Chemicals and Standards

A mix of certified reference standards at 100 µg mL^−1^ of tetracycline, oxytetracycline, ciprofloxacin, enrofloxacin, nalidixic acid, trimethoprim, sulfadiazine, erythromycin A, amoxicillin, ampicillin, penicillin G potassium salt, tylosin, cephalexin and streptomycin sulphate and another with meloxicam, flunixin, carprofen, ketoprofen, tolfenamic acid, diclofenac acid and phenylbutazone (CPA Chem, Stara Zagora, Bulgaria) in methanol were purchased from Cromlab (Barcelona, Spain). Ciprofloxacin-d8 hydrochloride hydrate (ref 32982) was purchased from Sigma Aldrich (Milan, Italy) and used as internal standard (IS). Methanol and acetonitrile residue quality (>99.9% purity) was obtained from Lab-Scan (Poland). For sample acidification, hydrochloric acid (HCl, Panreac, Spain) 37% diluted in double-distilled and deionised water until reaching a final concentration of 10% was used. Magnesium sulphate (MgSO_4_), sodium chloride (NaCl), sodium citrate dibasic sesquihydrate (NaCitrate dibasic sesquihydrate), sodium citrate tribasic dehydrate (NaCitrate tribasic dehydrate), PSA bonded silica (supelclean PSA: polymerically bonded, ethylenediamine-N-propyl phase with primary and secondary amines) and C18 (Discovery DSC-18: octadecylsilane 18% C) were purchased from Supelco (Bellefonte, PA, USA).

A standard mix containing every antibiotic and NSAID at 2000 ng mL^−1^ was made with an appropriate amount of methanol. This mix was used to spike the hen plasma samples for method validation.

### 2.3. Instruments and Conditions

To detect and quantify the pharmaceuticals, an HPLC system consisting of vacuum degasser, autosampler and binary pump (Agilent Series 1200, Agilent Technologies, Santa Clara, CA, USA) was used. Standards or samples (40 µL) were thermostated at 4 °C and injected onto a reversed phase rapid resolution Waters Sunfire C18 (4.6 × 150 mm, 5 µm) HPLC column at a flow rate of 0.8 mL/min. The column was equilibrated at 40 °C. In the case of positive ionization, solvents A (MilliQ water with 0.01% formic acid) and B (acetonitrile) were used for the compound separation. For negative ionization analysis, solvents A (MilliQ water with 5 mM ammonium acetate) and B (acetonitrile) were used. In both conditions, the elution program consisted on a linear gradient from 0% to 45% solvent B for 15 min; a linear gradient from 45% to 95% solvent B for 12 min; 95% solvent B, maintained for 3 min; initial condition (0% solvent B), applied for 3 min before the next injection. The total run time was 33 min. The HPLC system was connected to a time-of-flight mass spectrometer Agilent 6220 accurate mass TOF (Agilent Technologies, Santa Clara, CA) equipped with an electrospray interface operating in the positive and negative ionization mode, using the following operation parameters: nebulizer gas pressure, 60 psi; drying gas flow, 12 L/min at 350 °C in both conditions. The capillary spray, fragmentor and octopole voltages were 3500, 180 and 250 V, respectively, in positive ionization, and 3500, 180 and 250 V, respectively, in negative ionization. Profile data in the 100–1100 m/z range were acquired for MS scans in 4 GHz extended high-resolution mode with 2 spectra/s, 500 ms/spectrum and 9652 transients/spectrum. Reference masses at 121.0509 and 922.0098 were used for mass correction during the analysis in positive mode, whereas 112.9859 and 1033.9881 were used in negative mode

Full scan data were recorded with Agilent Mass Hunter Data Acquisition software (version B.06.00) and processed with Agilent Mass Hunter Qualitative Analysis software (version B.06.00, Service Pack 1, Agilent Technologies, Inc. USA, 2012). To identify the compounds, retention times of the analytes were compared with a standard compound (± 0.5 min), with the difference of the theoretical exact mass and the measured accurate masses of the analyte being ≤ 5 ppm. This information is provided in Table 1.

### 2.4. Sample Preparation

In the methods A, B and C, 2 mL of sample (whole blood or plasma) was homogenized with 2 mL of extraction solvent (acetonitrile or methanol). Method A used a mixture of whole blood and methanol, and method B used a mixture of whole blood and acetonitrile. Method C used a mixture of plasma and methanol. In each case, the mix was shaken vigorously with a vortex for 1 min followed by the addition of extraction salts (1.33 g MgSO_4_, 0.33 g NaCl, 0.17 g Na-citrate dibasic sesquihydrate and 0.33 g Na-citrate tribasic dehydrate) to separate the liquid phase and stabilize the compounds. For this, tubes were vigorously shaken again during 1 min with vortex. The tubes were centrifuged at 5000× *g* for 5 min (Sanyo MSE MISTRAL 2000 R) and kept at −20 °C for 1 h. Total supernatant was then purified by being transferred to another tube with 300 mg MgSO_4_, 50 mg PSA and 50 mg DSC-18. The tube was shaken similarly to the first step and then centrifuged again at 5000× *g* for 5 min. Total supernatant was transferred to a clean tube and evaporated until 1 mL under nitrogen stream to be transferred to a vial for HPLC analysis.

Method D was an adaptation of the simple extraction method developed previously for antibiotics [38]: 100 μL of plasma and 10 μL of IS were mixed with 10 μL of HCl (10%). This mixture was shaken vigorously with a vortex (VELP-Scientifica) for 5 s. Then, 280 μL was added, and the tube was shaken for 5 min with a vortex and left afterwards in ultrasounds bath (Selecta) for 5 min. The tube was then cooled for 5 min at −20 °C to contribute to proteins separation. Finally, the mix was centrifuged at 4 °C (Beckman, Microfuge-R) for another 5 min at 5000× *g*, and 200 μL of the supernatant was transferred to a vial and mixed with 200 μL of purified Milli-Q water before HPLC analysis.

### 2.5. Selection and Validation of the Optimal Extraction Technique

For the technique selection, accuracy and repeatability parameters were calculated using five replicates of spiked samples at 100 ng mL^−1^ that were processed by methods A, B, C and D. Validation of the modifications made to a method described previously [38] was based on the “Guidance Document on Analytical Quality Control and Method Validation Procedures for Pesticide Residues Analysis in Food and Geed from the Directorate-General for Health and Food Safety of the European Commission” [39], using four replicates for each level of spiked samples (25, 50, 100, 200 and 400 ng mL^−1^). These replicates were used to obtain a calibration curve by linear regression using the method of least squares, applied subsequently to calculate levels of pharmaceuticals in field samples. The acceptance criterion for linearity was a correlation coefficient *r* ≥ 0.9.

To determine the precision of a method, repeatability and reproducibility tests should be applied, calculating relative standard deviation (RSD) as follows: *RSD* (%) = (*SD*/*Xm*) × 100, where *SD* is the standard deviation of the whole series of measurements and *Xm* is the mean. RSD for both parameters should be ≤20% to accept the method. To calculate RSD of repeatability, four replicates of hen plasma samples spiked with the mix of pharmaceuticals at five levels (25, 50, 100, 200 and 400 ng mL^−1^) were analyzed. Reproducibility was calculated by analyzing four replicates of spiked hen plasma at 200 ng mL^−1^ with different analysts and on different days.

To assess accuracy, recoveries were calculated by analyzing four replicates of spiked hen plasma at five levels (25, 50, 100, 200 and 400 ng mL^−1^), comparing peak areas of the extracted spiked samples with peak areas obtained in the working standard solutions using the following formula: *Recovery* (%) = (*Cm*/*Cp*) × 100, where *Cm* is the average area of each compound in the sample and *Cp* is the area of each compound in the standard solution. Blank plasma samples were analyzed to ensure that they were free from the compounds analyzed and to test for a matrix effect.

Microsoft Excel 2016 was used to calculate the validation parameters of the technique.

## 3. Results

### 3.1. Method Validation

Four different protocols for extraction of veterinary drugs were tested. Three of them were based on a modification of QuEChERS, (Quick, Easy, Cheap, Effective, Rugged, and Safe) procedure extraction developed previously by Gómez-Ramírez et al. [26] (methods A, B and C, where sulfadiazine, nalidixic acid, trimethoprim, ciprofloxacin, enrofloxacin, tetracycline, oxytetracycline, flunixin, carprofen and meloxicam were detected); the last one was a simple extraction (method D) that detected tetracycline, oxytetracycline, ciprofloxacin, enrofloxacin, nalidixic acid, trimethoprim, sulfadiazine, meloxicam, flunixin, carprofen, tolfenamic acid and phenylbutazone. Although erythromycin A, amoxicillin, ampicillin, penicillin G potassium salt, tylosin, cephalexin, streptomycin sulphate, ketoprofen and diclofenac acid were also included in the mix of standards, they could not be detected in standard solution or in the spiked samples.

As shown in Table 2, recoveries obtained by QuEChERS methods A, B and C at 100 ng mL^−1^ were too low, being most of them below 60%. The greatest recovery (60.15%) was achieved by trimethoprim when we applied the QuEChERS method to plasma and used methanol as solvent. Moreover, the lowest recoveries were found in the case of method B, when acetonitrile was used as solvent.

**Figure 1:**
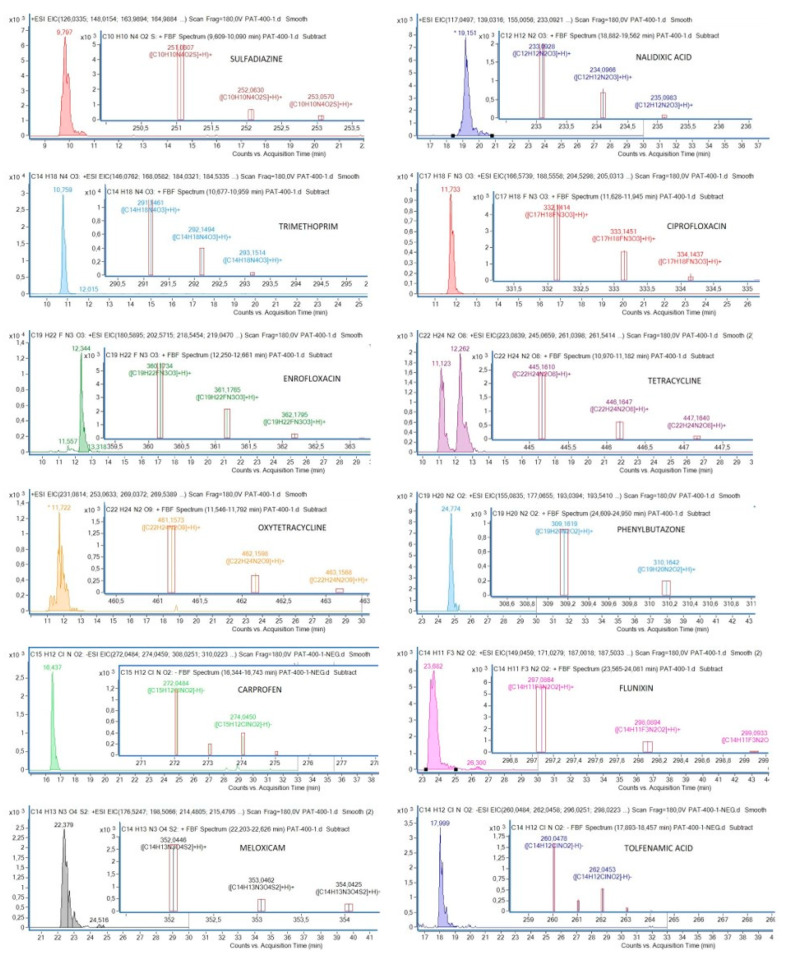
Chromatograms of each antibiotic and nonsteroidal anti-inflammatory drug (NSAID) and their mass spectra, detected by the validated method.

On the contrary, method D (simple extraction of plasma with methanol) provided very good recoveries for all the compounds at 100 ng mL^−1^ and for all the concentrations tested to validate the technique (Table 3). The high correlation coefficients, all above 0.98 except for carprofen (0.97), indicate very good correlations between concentrations of antibiotics and areas of the chromatographic peaks. Likewise, the method was considered precise, as RSD of repeatability and reproducibility were <20%, which is the threshold set to accept the validation of the analytical method [39]. Possible matrix effects may enhance or suppress ionization, leading to over- and under-recovery of some compounds. As shown in Table 3, recoveries for all the compounds were above 70%, except for meloxicam and carprofen at 25 ng mL^−1^ (30.65% and 52.38%, respectively). However, both compounds showed good linearity and precision values, which are needed to accept recoveries below 60% (but in any case >30%) in multiresidue methods [39]. The limit of quantification (LOQ), defined as the lowest concentration at which the analyte can be quantitated with a precision and accuracy of greater than 15%, was 25 ng mL^−1^ for all the compounds except carprofen and meloxicam (50 ng mL^−1^). Chromatograms of each antibiotic and NSAID detected by the validated method are shown in Figure 1.

### 3.2. Application to Field Samples from Wild Vultures

**Figure 2:**
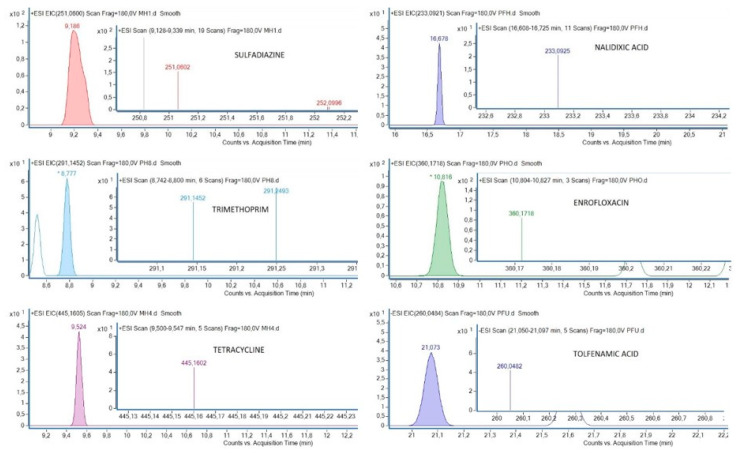
Chromatograms of some samples with the detected compounds.

Plasma of 29 griffon vulture nestlings was tested for traces of the pharmaceuticals listed above. As shown in Table 4, an antibiotic (enrofloxacin, 69.0%) and an NSAID (tolfenamic acid, 20.7%) were the pharmaceuticals most frequently detected in the vultures analyzed. All antibiotics except ciprofloxacin and oxytetracyclin were detected in at least one sample. However, in the case of NSAIDs, only tolfenamic acid was detected. Five out of the six individuals with residues of this NSAID also presented traces of enrofloxacin, while two of them also had a third compound (one with tetracycline and the other with nalidixic acid). Chromatograms of samples with the detected compounds are shown in Figure 2.

## 4. Discussion

### 4.1. Method Validation

According to the “Guidance Document on Analytical Quality Control and Method Validation Procedures for Pesticide Residues Analysis in Food and Feed from the Directorate-General for Health and Food Safety of the European Commission” [39], we achieved the validation of a miniaturized multiresidue method for quantifying antibiotics and NSAIDs of veterinary use using a very small sample volume. As mentioned above, sample amount is generally limited in studies of free-ranging wildlife, both by the weight of the individual sampled [29,30] and the need for other contaminant exposure assessment [31,32]. However, most analytical methods for quantifying veterinary drugs in complex matrices have been developed in the field of food safety [25], where sample amount of milk, meat or eggs is not a limiting factor, and often more than 2 g or 2 mL of sample are used [40]. This larger sample amount facilitates the analysis of xenobiotics, achieving lower LOQ and, in some cases, better validation parameters than in our method [25]. In fact, we had previously validated a method that achieved the quantification of a greater number of antibiotics, including β-lactams, using 2 g of tissue [26], and for this reason we sought to test it in plasma/blood samples (methods A, B and C).

Although the use of multiresidue methods to quantify veterinary drugs in small sample amounts (<1 g or mL) is becoming more popular [41], the number of studies is still limited, and they often focus on a particular group of compounds. For example, Barco et al. [42] validated a LC-MS method for analysis of 14 antibiotics (amikacin, amoxicillin, ceftazidime, ciprofloxacin, colistin, daptomycin, gentamicin, linezolid, meropenem, piperacillin, teicoplanin, tigecycline, tobramycin and vancomycin) and a β-lactamase inhibitor (tazobactam) using 100 µL of plasma from pediatric patients, obtaining good recoveries (>85%) and higher LOQ (100–2000 ng mL^−1^) than in our method. Similarly, Hermo et al. [43] also validated a method to analyze quinolones of veterinary use (marbofloxacin, ciprofloxacin, danofloxacin, enrofloxacin, sarafloxacin, difloxacin, oxolinic acid and flumequine) in bovine and porcine plasma using capillary electrophoresis and liquid chromatography with ultraviolet detection and liquid chromatography coupled with mass spectrometry and tandem mass spectrometry methods with 500 µL of plasma, reaching a LOQ of 2.5 ng mL^−1^. We anticipate that further improvements to our method will enable to quantification of other veterinary drugs (e.g., amoxicillin, diclofenac) to be achieved.

Although other mass spectrometry detectors coupled to HPLC have been commonly used to detect pharmaceuticals [41], MS-TOF detectors are becoming more popular, as they are more accurate than any other instrument due to the excellent ion separation and detection in the flight tube that provide a much better identification of target analytes in complex matrices such as food and animal tissues [44], including screening and confirmatory multiresidue analysis of veterinary drugs in food [41].

### 4.2. Application to Field Samples from Wild Vultures

Among all the antibiotics analyzed, enrofloxacin is the most commonly used for livestock, ranking as second most common product registered for veterinary use in Spain, after amoxicillin [45]. This quinolone was also detected in similar frequencies (69%) in a previous study in nestlings of the same species and study area sampled in 2013 (56%, *n* = 25) [6] and in full-grown vultures from Catalonia (67%, *n* = 61) and Navarra (64%, *n* = 45), Northern Spain, [17]. However, ciprofloxacin, the metabolite of enrofloxacin, was also detected and even quantified in both cited studies [6,17] and in other studies that determined the occurrence of quinolones in avian scavengers [13,15]. The lack of detection of ciprofloxacin in our samples may be explained by different factors. One can be related to the analytical method, since our method has been validated reaching higher LOQ (25 ng mL^−1^) than that used in other studies (5 ng mL^−1^ for methods used by Blanco et al. [6] and Casas-Díaz [17]). These studies also used greater sample volumes (0.5 and 0.2 mL, respectively) The timing of sampling is also relevant in the evaluation of exposure to xenobiotics with low half-lives in cattle and birds, such as quinolones (<10 h for enrofloxacin and <3 h for ciprofloxacin, [46]). Studies detecting ciprofloxacin in vultures may have sampled individuals that fed on livestock carrion at different time periods (earlier or later) than the individuals from our study. Hence, the vultures sampled in our study may have either already eliminated ciprofloxacin from the bloodstream or not metabolized it from enrofloxacin yet. However, according to pharmacokinetic studies reviewed by Martinez et al. [47], ∼50% of total enrofloxacin appears as ciprofloxacin in blood. This proportion between enrofloxacin and its metabolite ciprofloxacin has not always been found in wild vultures [13,17], although within-individual values of these quinolones were positively related in the Cinereous vulture (*Aegypius monachus*) [13]. Therefore, ciprofloxacin presence in blood of vultures can be an indicative of enrofloxacin ingestion, although it is not always possible to detect and correlate both compounds in free-living scavenging birds depending on the analytical method and the timing of feeding on medicated livestock carcasses. For these reasons, the analysis of metabolites, and not just the parent compounds, seems relevant for the assessment of exposure to pharmaceuticals in wildlife and is recommended.

Tolfenamic acid was the only NSAID detected in the samples, but the levels were below the LOQ. Its presence is not surprising, as it is used for medication of livestock, including pigs, in Spain [45]. To our knowledge, this is the first study to assess exposure to NSAIDs in free-living scavenger birds from Central Spain. In preliminary studies in adult griffon vultures captured in the Alcoy’s SFS (Alicante, Eastern Spain), flunixin was detected at high frequency, which was attributed to the vultures’ habit of feeding in groups on the same, contaminated, carcass [48]. To our knowledge, no study has evaluated the impact of tolfenamic acid on vulture health. Some recent reports (e.g., IUCN Vulture Specialist Group [49]) have identified this NSAID and meloxicam as a safe alternative to diclofenac. However, further studies including biomarkers of effects should be carried out.

To our knowledge, risk assessment studies concerning veterinary drugs in vultures have focused on a single group of compounds [6,13,16,17]. The simultaneous detection of an NSAID (tolfenamic acid) and several antibiotics (mainly enrofloxacin) indicates the vultures’ exposure to pharmaceutical mixtures, whose effects on their health deserve further research. This highlights the still scarce knowledge and need to investigate the occurrence of the residues of different compounds present within particular carcasses and accumulated in SFSs, which implies the use of multiresidue analytical methods.

## 5. Conclusions

The purpose of the validation of the analytical method has been fulfilled, since it allows the simultaneous quantification of low levels of seven antibiotics of different chemical classes and five NSAIDs in a single analysis, using a small volume of plasma.

To our knowledge, this is the first study to detail the exposure assessment of different classes of veterinary pharmaceuticals in scavenger birds with the simultaneous detection of antibiotics and NSAIDs in the same individuals.

## Figures and Tables

**Table 1:** List of compounds analyzed and their corresponding data (molecular formula, theoretical masses of their adducts) for identification in the HPLC-MS-TOF analysis in plasma samples.

Compound	Formula	Theoretical *m/z* H^+^	Theoretical *m/z* H^–^	∆ (ppm)
Sulfadiazine	C_10_H_10_N_4_O_2_S	251.06	-	3.59
Nalidixic acid	C_12_H_12_N_2_O_3_	233.093	-	3.53
Trimethoprim	C_14_H_18_N_4_O_3_	291.146	-	3.43
Ciprofloxacin	C_17_H_18_FN_3_O_3_	332.141	-	2.6
Enrofloxacin	C_19_H_22_FN_3_O_3_	360.172	-	2.72
Tetracycline	C_22_H_24_N_2_O_8_	445.161	-	3.53
Oxytetracycline	C_22_H_24_N_2_O_9_	461.156	-	3.44
Phenylbutazone	C_19_H_20_N_2_O_2_	309.160	-	4.4
Flunixin	C_14_H_11_F_3_N_2_O_2_	297.085	-	4
Carprofen	C_15_H_12_ClNO_2_	-	272.048	−1.51
Meloxicam	C_14_H_13_N_3_O_4_S_2_	352.042	-	5.6
Tolfenamic acid	C_14_H_12_ClNO_2_	-	260.048	−1.97

Δ (ppm) is the error of measurement of ion mass.

**Table 2:** Recoveries (%) obtained by the QuEChERS (Quick, Easy, Cheap, Effective, Rugged, and Safe) methods A, B and C at 100 ng mL^−1^.

	QuEChERS Extraction Method		
Pharmaceutical	A Whole Blood–Methanol	B Whole Blood–Acetonitrile	C Plasma–Methanol
Sulfadiazine	14.92	31.96	14.00
Nalidixic acid	59.87	1.87	47.82
Trimethoprim	56.34	43.15	60.16
Ciprofloxacin	50.12	0.05	49.64
Enrofloxacin	26.04	0.89	28.6
Tetracycline	11.11	0.28	15.37
Oxytetracycline	11.17	0.17	11.82
Flunixin	24.06	13.26	36.08
Carprofen	11.78	0.34	24.72
Meloxicam	28.82	27.22	43.94

**Table 3:** Accuracy, precision and linearity of the pharmaceuticals from spiked hen plasma samples.

	Recovery ^b^ (%)						Precision (RSD %)		Lin.^b^
Pharmaceutical	M ^a^	25	50	100	200	400	Rep. ^b^	Repr. ^c^	(*r*)
Sulfadiazine	101.03	106.03	100.06	108.13	96.94	94.01	5.97	5.61	0.983
Nalidixic acid	89.62	84.27	98.59	93.87	91.02	80.35	7.59	7.79	0.983
Trimethoprim	99.55	91.49	101.25	112.28	96.16	96.57	5.21	4.82	0.988
Ciprofloxacin	96.53	91.25	97.70	113.76	93.40	86.51	6.37	5.31	0.986
Enrofloxacin	105.56	100.10	108.10	113.19	100.01	106.40	6.91	7.56	0.990
Tetracycline	98.22	86.74	106.99	111.00	94.14	92.24	5.74	6.46	0.991
Oxytetracycline	97.78	96.16	97.63	106.02	96.26	92.83	7.36	8.22	0.988
Phenylbutazone	90.04	86.23	136.88	66.10	56.51	95.48	13.77	11.31	0.993
Flunixin	98.70	107.31	95.52	125.09	85.28	80.33	12.37	7.61	0.997
Carprofen	97.62	52.38	119.90	100.72	107.87	107.26	13.17	7.09	0.965
Meloxicam	79.25	30.65	68.70	100.22	104.00	92.67	14.11	19.39	0.992
Tolfenamic acid	114.92	172.60	144.57	77.81	88.08	91.53	10.92	4.72	0.998

***^a^*** Average recoveries of the five spiking levels; ***^b^*** Average of four replicates at five concentrations (25, 50, 100, 200 and 400 ng mL^−1^); ***^c^*** Average of four replicates at 200 ng mL^−1^; Rep. = repeatability; Repr. = reproducibility; Lin = linearity; *r* = regression coefficient.

**Table 4:** Frequency of detection and concentrations of antibiotics and NSAIDs in plasma of 29 griffon vulture nestlings.

Pharmaceutical	Frequency of Detection (Number of Samples with Residues)	Concentrations (ng mL^−1^) (min–max)
**Antibiotics**		
Sulfadiazine	3.45% (1)	< LOQ
Nalidixic acid	3.45% (1)	< LOQ
Trimethoprim	6.90 (2)	< LOQ
Ciprofloxacin	0%	-
Enrofloxacin	69.00% (20)	< LOQ
Tetracycline	3.45% (1)	1.73
Oxytetracycline	0%	-
**NSAIDs**		
Phenylbutazone	0%	-
Flunixin	0%	-
Carprofen	0%	-
Meloxicam	0%	-
Tolfenamic acid	20.70% (6)	7.95–11.22

< LOQ = detected below limit of quantification.

## Supplementary Materials

The following are available online at https://www.mdpi.com/1660-4601/17/11/4058/s1, Figure S1: Chromatograms of each antibiotic and NSAID and their mass spectra, detected by the validated method. Figure S2. Chromatograms of some samples with the detected compounds. Table S1 List of compounds analysed and their corresponding data for identification in the HPLC-TOFMS analysis in plasma samples: molecular formula, theoretical masses of their adducts. Δ ppm is the error of measurement of ion mass
