# Supplementary material for: Validation of Multi-Residue Method for Quantification of Antibiotics and NSAIDs in Avian Scavengers by Using Small Amounts of Plasma in HPLC-MS-TOF
Source: Int J Environ Res Public Health. 2020 Jun 6;17(11):4058. doi: 10.3390/ijerph17114058 (PMC7313014; doi:10.3390/ijerph17114058)
Supplement: Supplementary file 1 [file ijerph-17-04058-s001.pdf]

Supplementary Materials: The following are available online at www.mdpi.com/xxx/s1,

Figure S1: Chromatograms of each antibiotic and NSAID and their mass spectra, detected by the validated method.

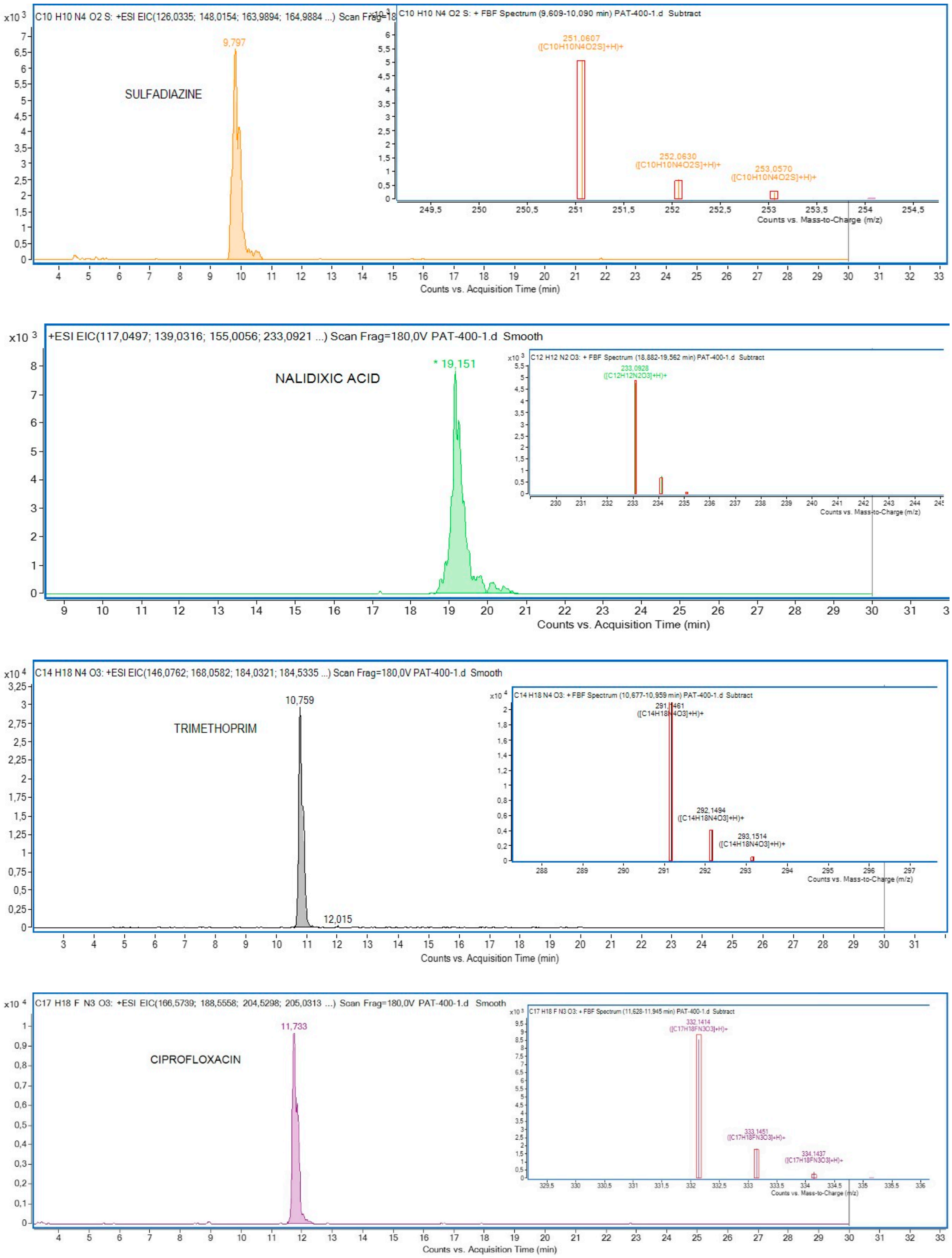

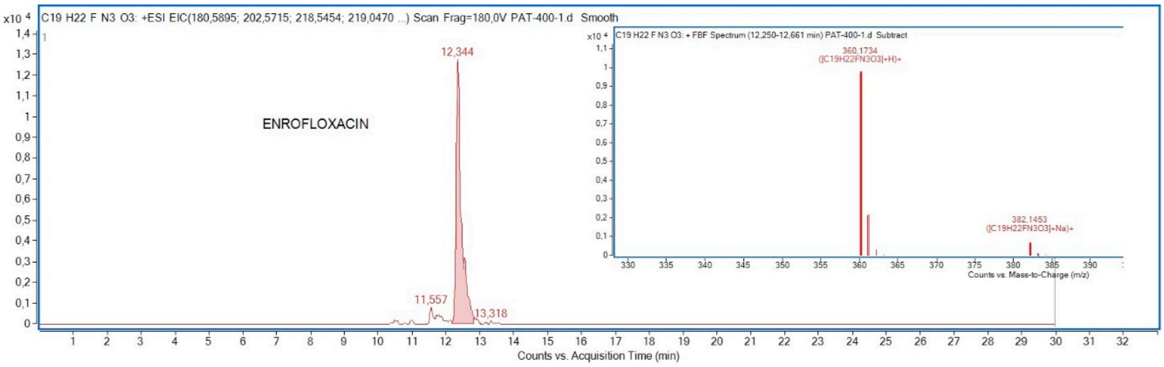
C19 H22 F N3 O3: +ESI EIC(180,5895; 202,5715; 218,5454; 219,0470 ...) Scan Frag=180,0V PAT-400-1.d Smooth
ENROFLOXACIN
12,344
11,557
13,318
Counts vs. Acquisition Time (min)
C19 H22 F N3 O3: + FBF Spectrum (12,250-12,661 min) PAT-400-1.d Subtract
360,1734
([C19H22FN3O3]+H)+
382,1453
([C19H22FN3O3]+Na)+
Counts vs. Mass-to-Charge (m/z)

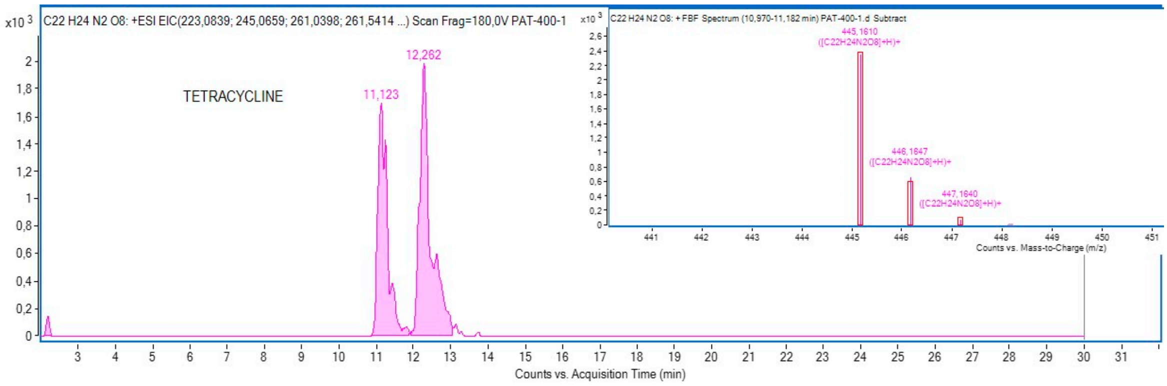
C22 H24 N2 O8: +ESI EIC(223,0839; 245,0659; 261,0398; 261,5414 ...) Scan Frag=180,0V PAT-400-1
TETRACYCLINE
11,123
12,262
Counts vs. Acquisition Time (min)
C22 H24 N2 O8: + FBF Spectrum (10,970-11,182 min) PAT-400-1.d Subtract
445,1610
([C22H24N2O8]+H)+
446,1647
([C22H24N2O8]+H)+
447,1640
([C22H24N2O8]+H)+
Counts vs. Mass-to-Charge (m/z)

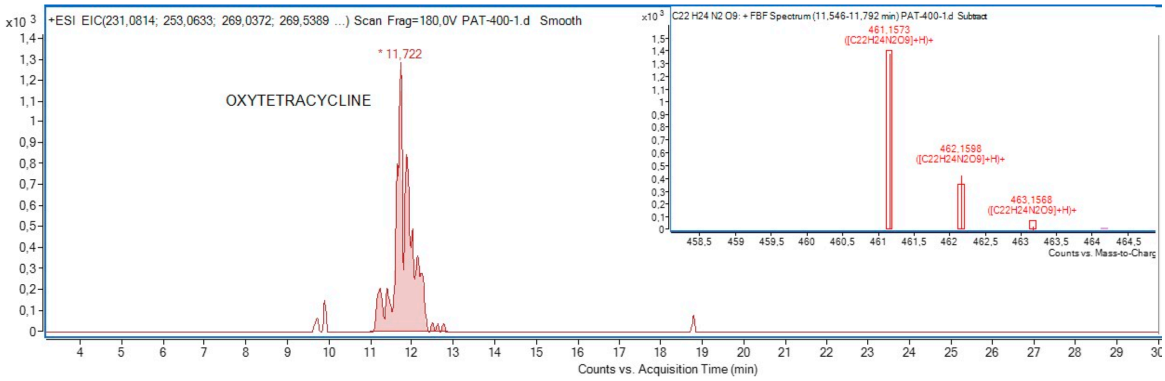
+ESI EIC(231,0814; 253,0633; 269,0372; 269,5389 ...) Scan Frag=180,0V PAT-400-1.d Smooth
OXYTETRACYCLINE
* 11,722
Counts vs. Acquisition Time (min)
C22 H24 N2 O9: + FBF Spectrum (11,546-11,792 min) PAT-400-1.d Subtract
461,1573
([C22H24N2O9]+H)+
462,1598
([C22H24N2O9]+H)+
463,1568
([C22H24N2O9]+H)+

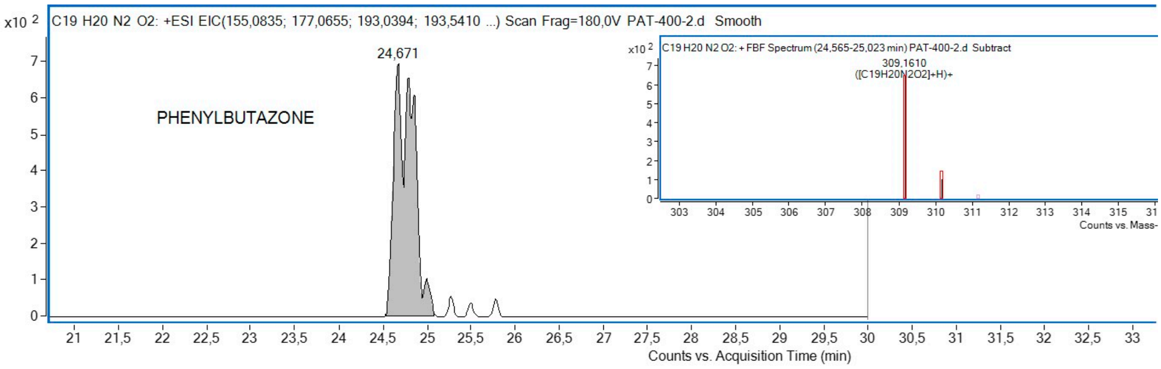
C19 H20 N2 O2: +ESI EIC(155,0835; 177,0655; 193,0394; 193,5410 ...) Scan Frag=180,0V PAT-400-2.d Smooth
PHENYLBUTAZONE
24,671
Counts vs. Acquisition Time (min)
C19 H20 N2 O2: + FBF Spectrum (24,565-25,023 min) PAT-400-2.d Subtract
309,1610
([C19H20N2O2]+H)+

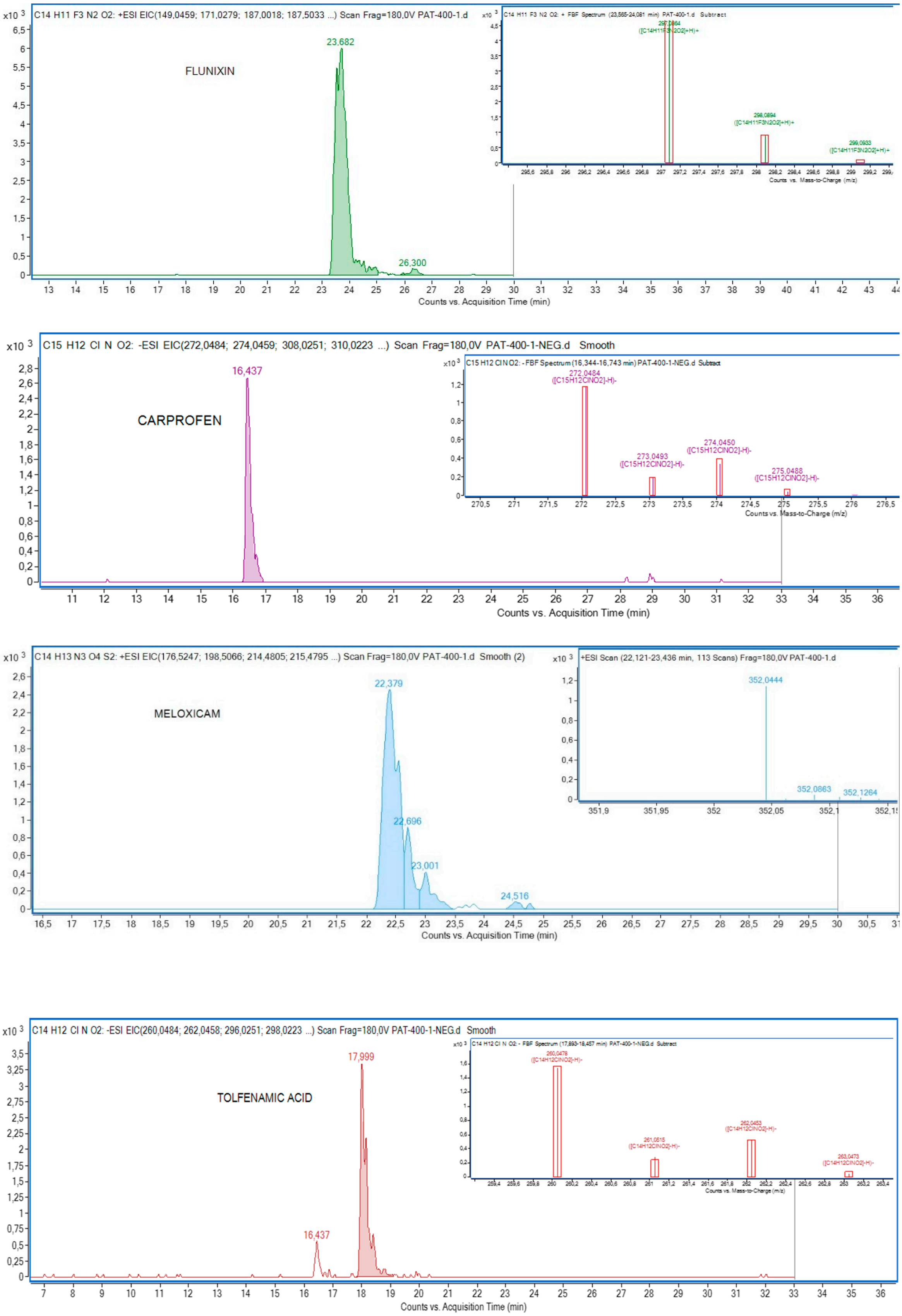

Figure S2. Chromatograms of some samples with the detected compounds.

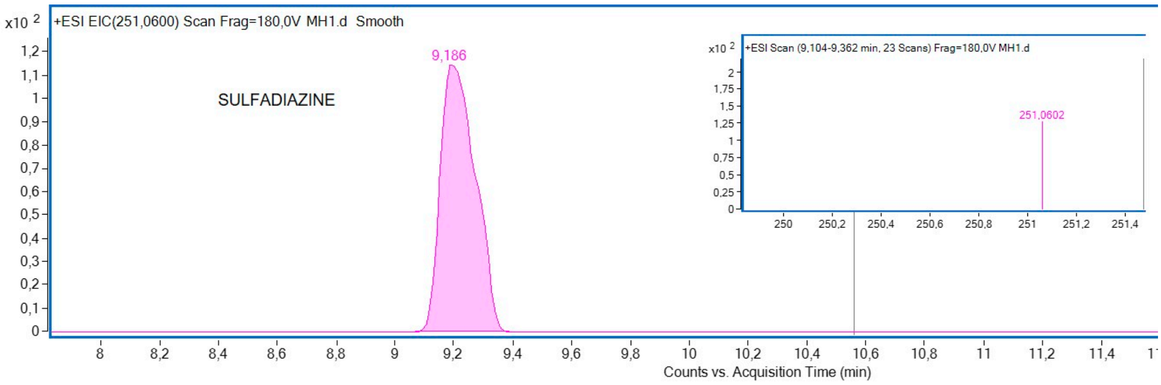
+ESI EIC(251,0600) Scan Frag=180,0V MH1.d Smooth
SULFADIAZINE
9,186
+ESI Scan (9,104-9,362 min, 23 Scans) Frag=180,0V MH1.d
251,0602
Counts vs. Acquisition Time (min)

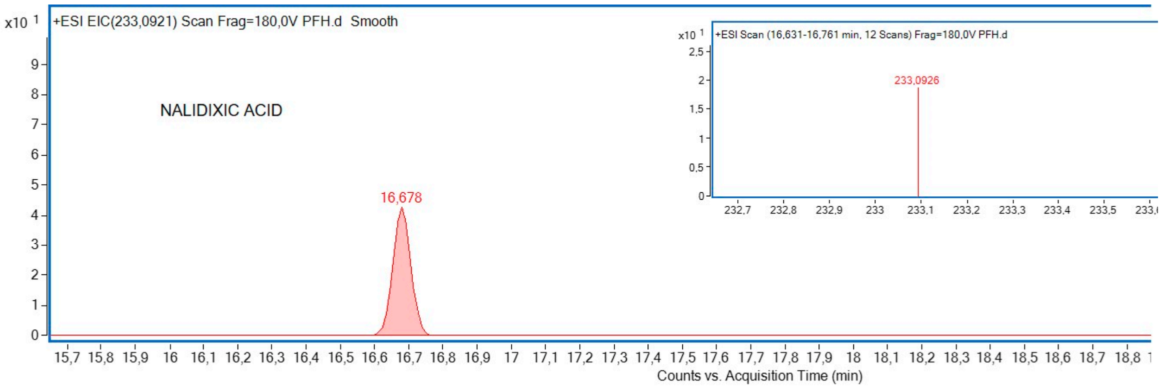
+ESI EIC(233,0921) Scan Frag=180,0V PFH.d Smooth
NALIDIXIC ACID
16,678
+ESI Scan (16,631-16,761 min, 12 Scans) Frag=180,0V PFH.d
233,0926
Counts vs. Acquisition Time (min)

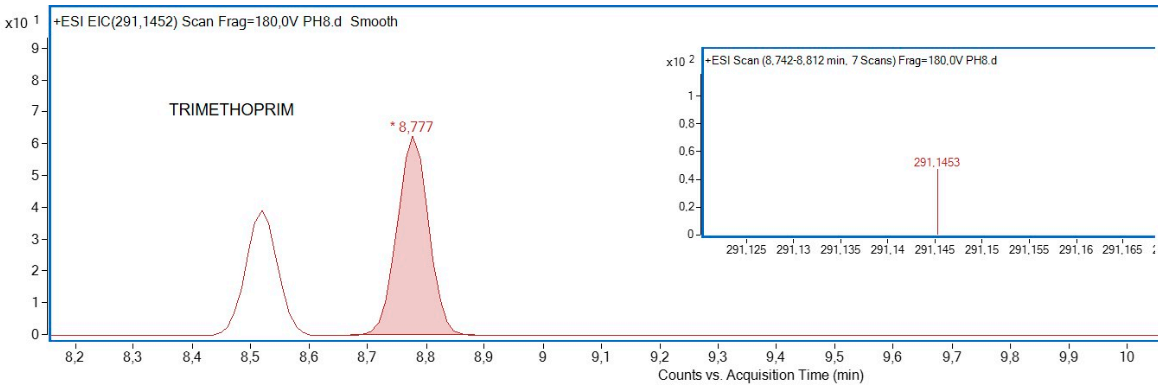
+ESI EIC(291,1452) Scan Frag=180,0V PH8.d Smooth
TRIMETHOPRIM
* 8,777
+ESI Scan (8,742-8,812 min, 7 Scans) Frag=180,0V PH8.d
291,1453
Counts vs. Acquisition Time (min)

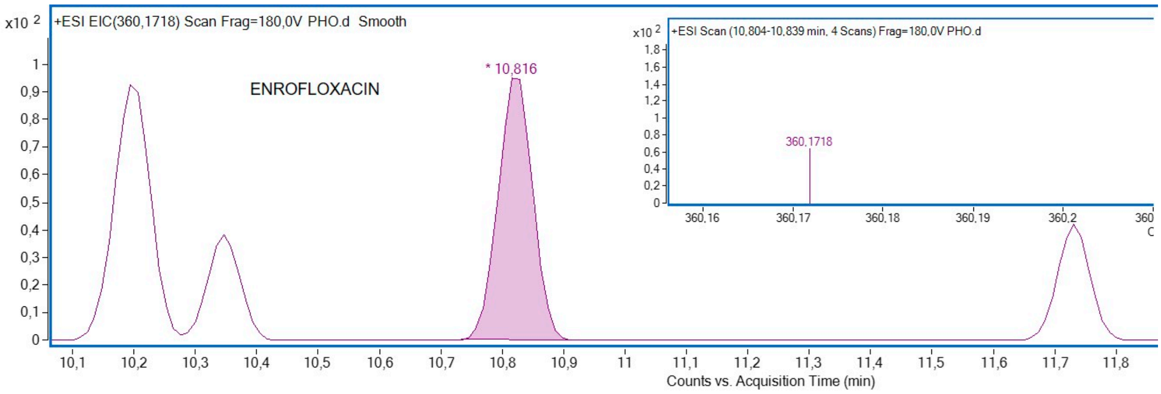
+ESI EIC(360,1718) Scan Frag=180,0V PHO.d Smooth
ENROFLOXACIN
* 10,816
+ESI Scan (10,804-10,839 min, 4 Scans) Frag=180,0V PHO.d
360,1718
Counts vs. Acquisition Time (min)

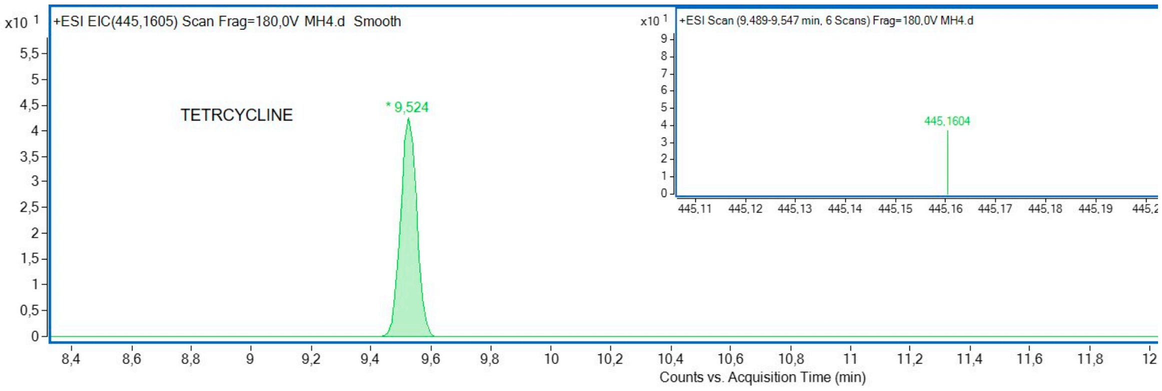

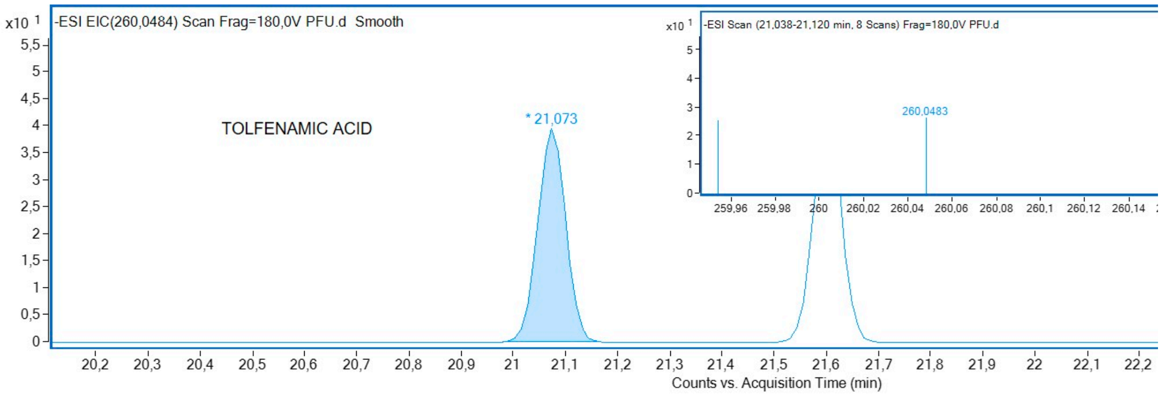

Table S1 List of compounds analysed and their corresponding data for identification in the HPLC-TOFMS analysis in plasma samples: molecular formula, theoretical masses of their adducts. Δ ppm is the error of measurement of ion mass

Compound	Formula	Theorethical *m/z* H+	Theorethical *m/z* H-	Δ (ppm)
Sulfadiazine	$C_{10}H_{10}N_4O_2S$	251.06	-	3.59
Nalidixic acid	$C_{12}H_{12}N_2O_3$	233.093	-	3.53
Trimethoprim	$C_{14}H_{18}N_4O_3$	291.146	-	3.43
Ciprofloxacin	$C_{17}H_{18}FN_3O_3$	332.141	-	2.6
Enrofloxacin	$C_{19}H_{22}FN_3O_3$	360.172	-	2.72
Tetracycline	$C_{22}H_{24}N_2O_8$	445.161	-	3.53
Oxytetracycline	$C_{22}H_{24}N_2O_9$	461.156	-	3.44
Phenylbutazone	$C_{19}H_{20}N_2O_2$	309.160	-	4.4
Flunixin	$C_{14}H_{11}F_3N_2O_2$	297.085	-	4
Carprofen	$C_{15}H_{12}ClNO_2$	-	272,048	-1.51
Meloxicam	$C_{14}H_{13}N_3O_4S_2$	352.042	-	5.6
Tolfenamic acid	$C_{14}H_{12}ClNO_2$	-	260,048	-1.97
